# 1-[2-Hy­droxy-4-(prop-2-yn-1-yl­oxy)phen­yl]ethanone

**DOI:** 10.1107/S1600536813032613

**Published:** 2013-12-07

**Authors:** V. Selvarani, M. A. Neelakantan, T. Srinivasan, D. Velmurugan

**Affiliations:** a Chemistry Research Centre, National Engineering College, K.R. Nagar, Kovilpatti 628 503, India; b Centre of Advanced Study in Crystallography and Biophysics, University of Madras, Guindy Campus, Chennai 600 025, India

## Abstract

In the title compound, C_11_H_10_O_3_, there is an intra­molecular O—H⋯O hydrogen bond generating an *S*(6) ring motif. The O atom of the hy­droxy group deviates by 0.0200 (1) Å from the benzene ring to which it is attached. The propyne group is almost linear, the C—C C angle being 177.83 (15)°, and is almost coplanar with the benzene ring; the C—C—O—C torsion angle being only −1.1 (2)°. In the crystal, mol­ecules are linked *via* C—H⋯O hydrogen bonds, forming infinite *C*(11) chains running parallel to [103]. These chains are linked by a pair of C—H⋯O hydrogen bonds, enclosing *R*_2_^2^(8) inversion dimers, forming a corrugated two-dimensional network lying parallel to (103).

## Related literature   

For the biological activity of benzaldehyde derivatives, see: Zhao *et al.* (2007 ▶); Ley & Bertram (2001 ▶); Delogu *et al.* (2010 ▶). For a related structure, see: Esakkiammal *et al.* (2012 ▶). For graph-set notation, see: Bernstein *et al.* (1995 ▶).
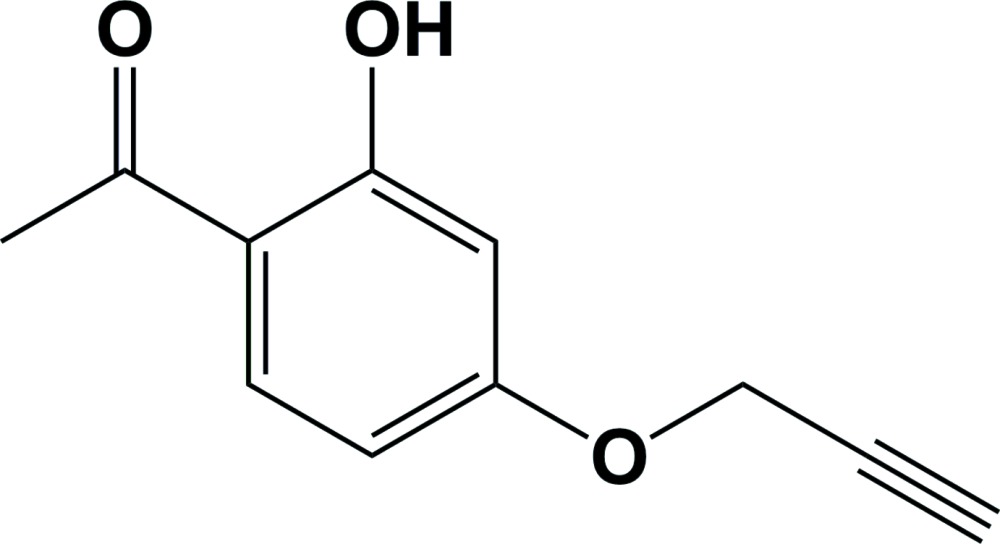


## Experimental   

### 

#### Crystal data  

C_11_H_10_O_3_
*M* *_r_* = 190.19
Monoclinic, 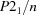

*a* = 4.9975 (2) Å
*b* = 10.4305 (4) Å
*c* = 18.8467 (7) Å
β = 97.257 (2)°
*V* = 974.54 (7) Å^3^
*Z* = 4
Mo *K*α radiation
μ = 0.10 mm^−1^
*T* = 293 K
0.35 × 0.30 × 0.20 mm

#### Data collection  

Bruker SMART APEXII area-detector diffractometer
Absorption correction: multi-scan (*SADABS*; Bruker, 2008 ▶) *T*_min_ = 0.968, *T*_max_ = 0.981
9316 measured reflections
2451 independent reflections
1898 reflections with *I* > 2σ(*I*)
*R*_int_ = 0.021

#### Refinement  

*R*[*F* ^2^ > 2σ(*F* ^2^)] = 0.043
*wR*(*F* ^2^) = 0.130
*S* = 1.05
2451 reflections
138 parameters
3 restraints
H atoms treated by a mixture of independent and constrained refinement
Δρ_max_ = 0.17 e Å^−3^
Δρ_min_ = −0.21 e Å^−3^

### 

Data collection: *APEX2* (Bruker, 2008 ▶); cell refinement: *SAINT* (Bruker, 2008 ▶); data reduction: *SAINT*; program(s) used to solve structure: *SHELXS97* (Sheldrick, 2008 ▶); program(s) used to refine structure: *SHELXL97* (Sheldrick, 2008 ▶); molecular graphics: *ORTEP-3 for Windows* (Farrugia, 2012 ▶) and *Mercury* (Macrae *et al.*, 2008 ▶); software used to prepare material for publication: *SHELXL97* and *PLATON* (Spek, 2009 ▶).

## Supplementary Material

Crystal structure: contains datablock(s) global, I. DOI: 10.1107/S1600536813032613/su2668sup1.cif

Structure factors: contains datablock(s) I. DOI: 10.1107/S1600536813032613/su2668Isup2.hkl

Supporting information file. DOI: 10.1107/S1600536813032613/su2668Isup3.cml

Additional supporting information:  crystallographic information; 3D view; checkCIF report

## Figures and Tables

**Table 1:** Hydrogen-bond geometry (Å, °)

*D*—H⋯*A*	*D*—H	H⋯*A*	*D*⋯*A*	*D*—H⋯*A*
O1—H1⋯O2	0.82	1.83	2.5551 (14)	146
C4—H4⋯O3^i^	0.93	2.54	3.4609 (16)	172
C11—H11⋯O2^ii^	0.93	2.32	3.2337 (18)	168

Symmetry codes: (i) 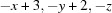; (ii) 
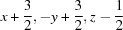
.

**Fig. 1.**
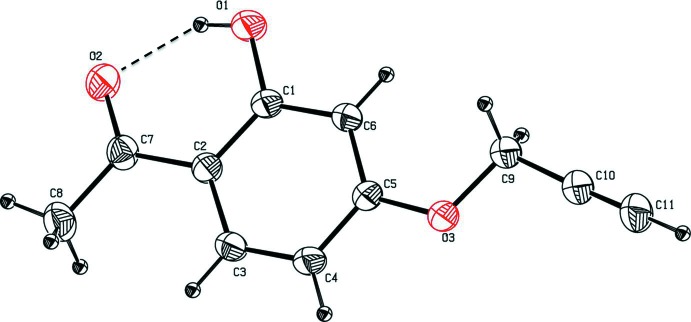
The molecular structure of the title molecule, with atom labelling. Displacement ellipsoids are drawn at the 30% probability level. The intramolecular O-H···O hydrogen bond is shown as a dashed line (see Table 1 for details)

**Fig. 2.**
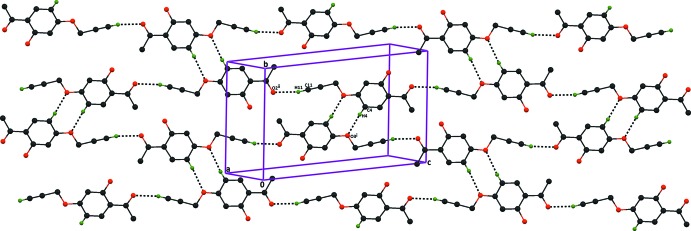
A view along the a axis of the crystal packing of the title compound. The hydrogen bonds are shown as dashed lines (see Table 1 for details; H atoms not involved in hydrogen bonding have been excluded for clarity).

## supplementary crystallographic information

### 1. Comment

Schiff bases derived from amines and substituted benzaldehydes exhibit antibacterial, anticancer and antitumour activities (Zhao *et al.*, 2007). Several benzaldoximes, benzaldehyde-O-ethyloximes and acetophenone oximes were synthesized and evaluated as tyrosinase inhibitors (Ley & Bertram, 2001). Bis-salicylaldehydes has been shown to exhibited greater inhibitory activity than salicylaldehyde (Delogu *et al.*, 2010). In view of these potential applications and in continuation of our work on the crystal structures of benzaldehyde derivatives, we synthesized the title compound and report herein on its crystal structure.

The molecular structure of the title compound is stabilized by an O—H···O intramolecular hydrogen bond (Fig. 1 and Table 1), which forms an S(6) graph-set motif (Bernstein *et al.*, 1995). The hydroxyl O atom, O1, deviates by 0.0200 (1) Å from the benzene ring (C1-C6) to which it is attached. The oxygen atom substituted propyne group is slightly twisted from the benzene ring (C1–C6) to which it is attached as evidenced by the torsion angle C6–C5–O3–C9 = -1.1 (2) °. The propyne group is almost linear, the C9–C10≡C11 angle being 177.83 (15)°, and it is also in the flagpole position on atom O3. The mean plane of the acetaldehyde group makes a dihedral angle of 0.39 (9)° with the benzene ring (C1–C6), indicating that they are almost coplanar.

In the crystal, molecules are linked via C—H···O hydrogen bonds forming infinite C(11) chains running parallel to direction [103]. These chains are linked via a pair of C—H···O hydrogen bonds, enclosing R_2_^2^(8) inversion dimers, forming wave-like two-dimensional networks lying parallel to (103); see Table 1 and Fig. 2.

### 2. Experimental

Equimolar amounts of 3-bromopropyne (10 mmol), 2,4-dihydroxyacetophenone (10 mmol) and potassium carbonate (15 mmol) were suspended in dried acetone (30 ml) and refluxed for 5 h. The reaction mixture was filtered while hot to remove insoluble impurities, neutralized with water and then extracted with ethyl acetate and dried with Na_2_SO_4_. The extracts were concentrated to obtain a brown solid which was then purified by column chromatography over SiO_2_ by eluting with a mixture of 5% ethyl acetate in n-hexane. Evaporation of the purified extract yielded the title compound in the form of a pure white solid [Yield: 83%]. Colourless block-like crystals, suitable for X-ray diffraction analysis, were obtained by the slow evaporation of a solution in ethyl acetate.

### 3. Refinement

All H atoms could be located in difference Fourier maps. The methyl H atoms were refined with U_iso_(H) = 1.5U_eq_(C). The OH and other C-bound H atoms were included in calculated positions are refined as riding atoms: O-H = 0.82 Å, C-H = 0.93 and 0.97 Å for CH and CH_2_ H atoms, respectively, with U_iso_(H) = 1.5U_eq_(O) and = 1.2U_eq_(C) for other H atoms.

### Crystal data

**Table:**

C_11_H_10_O_3_	*F*(000) = 400
*M**_r_* = 190.19	*D*_x_ = 1.296 Mg m^−^^3^
Monoclinic, *P*2_1_/*n*	Mo *K*α radiation, λ = 0.71073 Å
Hall symbol: -P 2yn	Cell parameters from 2451 reflections
*a* = 4.9975 (2) Å	θ = 2.2–28.4°
*b* = 10.4305 (4) Å	µ = 0.10 mm^−^^1^
*c* = 18.8467 (7) Å	*T* = 293 K
β = 97.257 (2)°	Block, colourless
*V* = 974.54 (7) Å^3^	0.35 × 0.30 × 0.20 mm
*Z* = 4	

### Data collection

**Table:**

Bruker SMART APEXII area-detector diffractometer	2451 independent reflections
Radiation source: fine-focus sealed tube	1898 reflections with *I* > 2σ(*I*)
Graphite monochromator	*R*_int_ = 0.021
ω and φ scans	θ_max_ = 28.4°, θ_min_ = 2.2°
Absorption correction: multi-scan (*SADABS*; Bruker, 2008)	*h* = −6→6
*T*_min_ = 0.968, *T*_max_ = 0.981	*k* = −13→13
9316 measured reflections	*l* = −25→25

### Refinement

**Table:**

Refinement on *F*^2^	Secondary atom site location: difference Fourier map
Least-squares matrix: full	Hydrogen site location: inferred from neighbouring sites
*R*[*F*^2^ > 2σ(*F*^2^)] = 0.043	H atoms treated by a mixture of independent and constrained refinement
*wR*(*F*^2^) = 0.130	*w* = 1/[σ^2^(*F*_o_^2^) + (0.0637*P*)^2^ + 0.1394*P*] where *P* = (*F*_o_^2^ + 2*F*_c_^2^)/3
*S* = 1.05	(Δ/σ)_max_ < 0.001
2451 reflections	Δρ_max_ = 0.17 e Å^−^^3^
138 parameters	Δρ_min_ = −0.21 e Å^−^^3^
3 restraints	Extinction correction: *SHELXL97* (Sheldrick, 2008), Fc^*^=kFc[1+0.001xFc^2^λ^3^/sin(2θ)]^-1/4^
Primary atom site location: structure-invariant direct methods	Extinction coefficient: 0.020 (4)

### Special details

**Table:**

Geometry. All esds (except the esd in the dihedral angle between two l.s. planes) are estimated using the full covariance matrix. The cell esds are taken into account individually in the estimation of esds in distances, angles and torsion angles; correlations between esds in cell parameters are only used when they are defined by crystal symmetry. An approximate (isotropic) treatment of cell esds is used for estimating esds involving l.s. planes.
Refinement. Refinement of F^2^ against ALL reflections. The weighted R-factor wR and goodness of fit S are based on F^2^, conventional R-factors R are based on F, with F set to zero for negative F^2^. The threshold expression of F^2^ > 2sigma(F^2^) is used only for calculating R-factors(gt) etc. and is not relevant to the choice of reflections for refinement. R-factors based on F^2^ are statistically about twice as large as those based on F, and R- factors based on ALL data will be even larger.

### Fractional atomic coordinates and isotropic or equivalent isotropic displacement parameters (Å^2^)

**Table:**

	*x*	*y*	*z*	*U*_iso_*/*U*_eq_	
C1	0.9425 (3)	0.69719 (11)	0.08613 (7)	0.0455 (3)	
C2	0.9120 (2)	0.81055 (10)	0.12463 (6)	0.0421 (3)	
C3	1.0579 (3)	0.91806 (11)	0.10720 (6)	0.0475 (3)	
H3	1.0417	0.9941	0.1320	0.057*	
C4	1.2229 (3)	0.91474 (11)	0.05494 (7)	0.0506 (3)	
H4	1.3186	0.9874	0.0446	0.061*	
C5	1.2467 (3)	0.80135 (11)	0.01728 (6)	0.0446 (3)	
C6	1.1094 (3)	0.69245 (11)	0.03266 (6)	0.0468 (3)	
H6	1.1283	0.6169	0.0076	0.056*	
C7	0.7340 (3)	0.81464 (12)	0.18017 (6)	0.0485 (3)	
C8	0.7041 (4)	0.93600 (15)	0.22030 (9)	0.0632 (4)	
H8A	0.595 (4)	0.9292 (18)	0.2554 (11)	0.095*	
H8B	0.648 (4)	1.005 (2)	0.1896 (10)	0.095*	
H8C	0.872 (4)	0.9636 (19)	0.2446 (10)	0.095*	
C9	1.4462 (3)	0.69606 (12)	−0.07557 (7)	0.0527 (3)	
H9A	1.2727	0.6639	−0.0972	0.063*	
H9B	1.5342	0.6298	−0.0449	0.063*	
C10	1.6120 (3)	0.73056 (13)	−0.13064 (7)	0.0546 (3)	
C11	1.7405 (3)	0.75594 (16)	−0.17657 (8)	0.0680 (4)	
H11	1.8424	0.7761	−0.2130	0.082*	
O1	0.8072 (2)	0.58946 (8)	0.09853 (6)	0.0691 (4)	
H1	0.7187	0.6017	0.1317	0.104*	
O2	0.6074 (2)	0.71799 (10)	0.19459 (5)	0.0624 (3)	
O3	1.4107 (2)	0.80891 (8)	−0.03494 (5)	0.0574 (3)	

### Atomic displacement parameters (Å^2^)

**Table:**

	*U*^11^	*U*^22^	*U*^33^	*U*^12^	*U*^13^	*U*^23^
C1	0.0506 (7)	0.0372 (5)	0.0508 (6)	−0.0075 (5)	0.0149 (5)	0.0001 (4)
C2	0.0460 (6)	0.0395 (5)	0.0418 (5)	−0.0015 (5)	0.0098 (5)	0.0009 (4)
C3	0.0570 (7)	0.0372 (5)	0.0501 (6)	−0.0056 (5)	0.0140 (5)	−0.0060 (4)
C4	0.0595 (8)	0.0385 (6)	0.0569 (7)	−0.0126 (5)	0.0196 (6)	−0.0035 (5)
C5	0.0457 (7)	0.0431 (6)	0.0472 (6)	−0.0071 (5)	0.0149 (5)	−0.0022 (4)
C6	0.0531 (7)	0.0370 (5)	0.0531 (6)	−0.0072 (5)	0.0177 (6)	−0.0073 (4)
C7	0.0549 (8)	0.0484 (6)	0.0438 (6)	0.0027 (5)	0.0127 (5)	0.0033 (5)
C8	0.0781 (11)	0.0589 (8)	0.0572 (8)	0.0039 (8)	0.0270 (8)	−0.0065 (6)
C9	0.0567 (8)	0.0491 (7)	0.0558 (7)	−0.0084 (6)	0.0200 (6)	−0.0096 (5)
C10	0.0569 (8)	0.0544 (7)	0.0553 (7)	−0.0037 (6)	0.0179 (6)	−0.0085 (6)
C11	0.0790 (11)	0.0666 (9)	0.0649 (8)	−0.0040 (8)	0.0339 (8)	−0.0095 (7)
O1	0.0912 (8)	0.0423 (5)	0.0835 (7)	−0.0214 (5)	0.0488 (6)	−0.0082 (4)
O2	0.0751 (7)	0.0571 (6)	0.0612 (6)	−0.0077 (5)	0.0334 (5)	0.0043 (4)
O3	0.0673 (6)	0.0475 (5)	0.0644 (6)	−0.0165 (4)	0.0354 (5)	−0.0110 (4)

### Geometric parameters (Å, º)

**Table:**

C1—O1	1.3469 (14)	C7—O2	1.2384 (15)
C1—C6	1.3879 (16)	C7—C8	1.4918 (18)
C1—C2	1.4056 (16)	C8—H8A	0.91 (2)
C2—C3	1.3991 (16)	C8—H8B	0.94 (2)
C2—C7	1.4573 (16)	C8—H8C	0.95 (2)
C3—C4	1.3627 (17)	C9—O3	1.4275 (14)
C3—H3	0.9300	C9—C10	1.4529 (18)
C4—C5	1.3920 (16)	C9—H9A	0.9700
C4—H4	0.9300	C9—H9B	0.9700
C5—O3	1.3602 (14)	C10—C11	1.1712 (19)
C5—C6	1.3765 (16)	C11—H11	0.9300
C6—H6	0.9300	O1—H1	0.8200
			
O1—C1—C6	117.20 (10)	O2—C7—C8	119.53 (12)
O1—C1—C2	121.51 (11)	C2—C7—C8	119.89 (11)
C6—C1—C2	121.27 (10)	C7—C8—H8A	114.1 (12)
C3—C2—C1	117.34 (11)	C7—C8—H8B	112.2 (12)
C3—C2—C7	121.92 (10)	H8A—C8—H8B	110.2 (17)
C1—C2—C7	120.75 (10)	C7—C8—H8C	111.3 (12)
C4—C3—C2	122.03 (10)	H8A—C8—H8C	104.4 (16)
C4—C3—H3	119.0	H8B—C8—H8C	103.9 (17)
C2—C3—H3	119.0	O3—C9—C10	107.45 (10)
C3—C4—C5	119.24 (10)	O3—C9—H9A	110.2
C3—C4—H4	120.4	C10—C9—H9A	110.2
C5—C4—H4	120.4	O3—C9—H9B	110.2
O3—C5—C6	124.19 (10)	C10—C9—H9B	110.2
O3—C5—C4	114.68 (10)	H9A—C9—H9B	108.5
C6—C5—C4	121.13 (10)	C11—C10—C9	177.83 (15)
C5—C6—C1	118.99 (10)	C10—C11—H11	180.0
C5—C6—H6	120.5	C1—O1—H1	109.5
C1—C6—H6	120.5	C5—O3—C9	117.87 (9)
O2—C7—C2	120.59 (11)		
			
O1—C1—C2—C3	−179.06 (12)	C4—C5—C6—C1	0.8 (2)
C6—C1—C2—C3	−0.40 (19)	O1—C1—C6—C5	178.60 (12)
O1—C1—C2—C7	0.7 (2)	C2—C1—C6—C5	−0.1 (2)
C6—C1—C2—C7	179.39 (12)	C3—C2—C7—O2	−179.90 (12)
C1—C2—C3—C4	0.3 (2)	C1—C2—C7—O2	0.3 (2)
C7—C2—C3—C4	−179.52 (12)	C3—C2—C7—C8	−0.1 (2)
C2—C3—C4—C5	0.4 (2)	C1—C2—C7—C8	−179.89 (13)
C3—C4—C5—O3	178.37 (12)	C6—C5—O3—C9	−1.1 (2)
C3—C4—C5—C6	−0.9 (2)	C4—C5—O3—C9	179.62 (12)
O3—C5—C6—C1	−178.44 (12)	C10—C9—O3—C5	175.97 (12)

### Hydrogen-bond geometry (Å, º)

**Table:**

*D*—H···*A*	*D*—H	H···*A*	*D*···*A*	*D*—H···*A*
O1—H1···O2	0.82	1.83	2.5551 (14)	146
C4—H4···O3^i^	0.93	2.54	3.4609 (16)	172
C11—H11···O2^ii^	0.93	2.32	3.2337 (18)	168

Symmetry codes: (i) −*x*+3, −*y*+2, −*z*; (ii) *x*+3/2, −*y*+3/2, *z*−1/2.
